# Antibiotic use for empirical therapy in the critical care units in primary and secondary hospitals in Vietnam: a multicenter cross-sectional study

**DOI:** 10.1016/j.lanwpc.2021.100306

**Published:** 2021-11-03

**Authors:** Vu Quoc Dat, Tran Tat Dat, Vu Quang Hieu, Kim Bao Giang, Satoko Otsu

**Affiliations:** a Department of Infectious Diseases, Hanoi Medical University, Hanoi, Vietnam (No 1 Ton That Tung street, Dong Da district, Hanoi, Vietnam); b Hanoi Medical University Hospital, Hanoi Medical University, Hanoi, Vietnam (No 1 Ton That Tung street, Dong Da district, Hanoi, Vietnam); c World Health Organisation Viet Nam Country Office, Hanoi, VietNam (304 Kim Ma Street, Hanoi, VietNam); d Institute for Preventive Medicine and Public Health, Hanoi Medical University, Hanoi, Vietnam (No 1 Ton That Tung street, Dong Da district, Hanoi, Vietnam); e Infectious Disease Department, Japanese Red Cross Wakayama Medical Centre, Wakayama City, Wakayama, Japan (4-20 Komatsubara-dori, Wakayama City 640-8558, Wakayama, Japan)

**Keywords:** antibiotic, antimicrobials, AWaRe, critical care, emergency, severe acute respiratory, infection, ICD-10, sepsis

## Abstract

**Background:**

The high rate of infections among patients admitted to critical care units (CCUs) is associated with high rate of antibiotic consumption, especially broad-spectrum antibiotics. This study is to describe the antibiotics use in CCUs in primary and secondary hospitals in Vietnam, a setting with high burden of antibiotic resistance.

**Methods:**

This was a 7-day observational study in 51 CCUs in hospitals from 5 provinces in Vietnam from March to July 2019. Patients aged ≥ 18 years admitted to the participating CCUs was enrolled consecutively. We collected data on patient's demographics, initial diagnosis and antibiotic therapy within the first 24 hours. Antibiotic therapy was classified by the Anatomical Therapeutic Chemical (ATC) Index and the 2019 WHO Access, Watch, Reserve (AWaRe) groups.

**Findings:**

Out of 1747 enrolled patients, empirical antibiotic treatments were initiated in 1112 (63.6%) patients. The most frequently prescribed antibiotics were cefotaxime (22.3%), levofloxacin (19%) and ceftazidime (10.8%). Antibiotics were given in 31.5% of patients without diagnosis of infection. Watch and/or Reserve group antibiotic were given in 87.3% of patients and associated with patient's age (aOR 1.01 per 1-year increment, 95%CI 1.00-1.02) and the presence of SIRS on admission (aOR 2.1, 95%CI 1.38-3.2).

**Interpretation:**

We observed a high frequency use and a substantial variation in patterns of empirical antibiotic use in the CCUs in Vietnam. It highlights the importance of continuous monitoring antibiotic consumption in CCUs.

**Research in context**

**Evidence before this study**

We searched in the Pubmed between August 1, 2016 and August 1, 2021 with the terms “AWaRe classification” or “AWaRe”, “antibiotic” or “antimicrobials” and “Vietnam”. Only two studies evaluated the antibiotic purchases in community and in hospitals using the WHO Access, Watch, Reserve (AWaRe) classification. No study of has been performed specifically to evaluate the empiric antibiotic treatments in patients admitted to the critical care units (CCUs) in Vietnam up to the time of our study.

**Added value of this study**

This study is the first to explore the empirical antibiotic treatments in critical care units in Vietnam where the prevalence of antimicrobial resistance is high. Furthermore, we suggest that the AWaRe classification is a simple indicator for antibiotic use in the CCUs and could be used to monitor or evaluate empirical antibiotic use for different clinical diagnosis and syndrome.

**Implications of all the available evidence**

Our study showed that it is feasible to monitor the patterns of antibiotic use in CCUs using the AWaRe classification. Compliance with guidelines on antibiotic therapy should be further evaluated in stewardship programmes.

Alt-text: Unlabelled box

## Introduction

Severe acute respiratory infection (SARI) and sepsis are leading causes of mortality worldwide with more than 336 million episodes of SARI in 2106 and more than 19 million people with sepsis annually.^1^^,^^2^ Delay in the initiation of appropriate antibiotic therapy for suspected bacterial infections is associated with an increase in adverse outcomes including death.^3^^,^^4^ Selection of empiric antibiotics depends on patient's characteristics, suspected site of infections, differential diagnosis, local microbial susceptibility data and antibiotic stewardship. Other consideration of empirical therapy may include the cost of treatment, availability of antibiotics, potential drug intolerances and toxicity.^5^

With a high burden of infectious diseases, critical care units had the highest consumption of antibiotics with an estimate of 71% of patients receiving any antibiotic.^6^ However, the rate of inappropriate antibiotic prescription in this setting may be up to one third.^7^ Beyond the benefit of early empiric antibiotic therapy to improve the outcome in patient with sepsis and septic shock,^8^ the use of broad-spectrum antibiotics is associated with adverse clinical effects, including destruction of the normal gut microbiota and development and selection of multidrug resistance organisms.^9^ The accurate diagnosis of infection in intensive care unit (ICU) is challanging and up to 43% of patients were treated inappropriately for infection.^10^ High selective pressure favouring resistant bacteria is exerted by the intensive and frequent antibiotic use.^11^^,^^12^

WHO first released a global report on the consumption of antibiotics from 65 countries and areas in 2016 and the most updated report was release on 2018.^13^^,^^14^ However, many consumption data is the lack of information on how antibiotics are prescribed and used at the patient level and in critical care setting. The gap in use of antibiotics should be addressed by antibiotic stewardship programme. The AWaRe classification of antibiotics (Access, Watch and Reserve groups) was first introduced in 2017 and revised in 2019 to promote antibiotic stewardship and address the challenge of increased antibiotic resistance.^14^ It is a transparent tool to track and measure the consumption of antibiotics at local, national and global level and easy use for monitoring the antibiotic use.

Vietnam has one of the highest potential of antibiotic drug resistance in Asia.^15^ During the period of 2013 to 2016, the resistance to third-generation cephalosporin in *E. coli* increased from 64% to 71%, the resistance to carbapenem in *K. pneumonia* increased from 23% to 24% and the proportion of methicillin-resistant *Staphylococcus aureus* (MRSA) increased from 46% to 73%.^16^ Because of the high rate of resistant bacteria, timeliness of empiric therapy is still important for critically ill patients and therefore, it is challenging to choose empirical antibiotics for severe life-threatening infections in CCUs. There is limited data on antibiotic initiation in CCUs in Vietnam. This study aims to describe the current situation of empirical antibiotic therapy in patients admitted to CCUs in Vietnam.

## Methods

### Study design and data collection

Viet Nam is composed of 63 provinces, including five centrally governed cities (Ha Noi, Ho Chi Minh City, Can Tho, Da Nang and Hai Phong). Administrative divisions of Viet Nam is consisted with province, district, and commune and each level has health care facilities according to their capacity (e.g. provincial hospital, district hospital, and commune health centre), in addition to national hospital in the central cities. This is the layer of reference system, e.g. a district hospital refer to a provincial hospital, and the national hospital. In this manuscript, ‘primary and secondary CCUs’ refers to CCUs in district or provincial hospitals.

We conducted a cross-sectional study in 2 centrally governed cities (Hanoi and Can Tho) and 3 provinces (Hanam, Thai Nguyen, Kontum) in 5 ecological regions in Vietnam from March to July 2019. In each province, we invited all CCUs in primary and secondary hospitals to participate in a 7-day prospective, observational cohort study in patients presented to critical care units. All patients admitted at the CCUs in selected hospitals for 7 days from the study initiation were included in this study. We collected the information of demographics, diagnosis, antibiotic prescriptions and data derived severity scores within 24 hours of admission and the outcomes at 7 days after the admission. Data was extracted from the medical charts. Doctors were not informed about the contents of the analysis and they managed their patients as they would normally.

### Study definitions

The initial diagnosis on admission to the CCUs were defined by the International Statistical Classification of Diseases and Related Health Problems 10th Revision (ICD-10)- WHO Version 2019. The diagnosis was made by the treating doctors in both ICD code and free text descriptions as a routine practise. All diagnosis were further cleaned and verified by two study doctors (VQD and TTD) by comparison of the consistency between ICD-10 coding and free-text data of diagnosis and severity across the patients and study sites. Discrepancies between the code and free texts were resolved by consensus by two study doctors. Patients were further defined as having severe acute respiratory infections (SARI) if they had a registered ICD-10 diagnosis code of J00-J06 (acute upper respiratory infections), J09-J18 (influenza and pneumonia) and J20-J22 (other acute respiratory infections)^17^ or an ICD-10 diagnosis code of J44.1 (exacerbation of chronic obstructive pulmonary disease, COPD). Other infections were defined in individuals who did not meet the SARI definition but had at least one diagnosis of corresponding ICD-10 codes for the remaining miscellaneous infections.

We used the 2019 WHO AWaRe classification of antibiotics to describe the empirical antibiotic therapy in patients presenting to the CCUs.^14^ The Access group includes first and second choice antibiotics for the empirical treatment of ubiquitous pathogens which should be widely applicable in all healthcare circumstances. The Watch group includes antibiotic classes that have higher risk of antibiotic resistance and are recommended for a limited number of indications. Lastly, the Reserve group includes antibiotics that are highly recommended for patients with multi-drug-resistant organisms.^14^

Systemic inflammatory response syndrome (SIRS) was defined in patients with at least two of the following criteria within 48 hours of admission to CCUs: body temperature >38.0°C or <36.0°C, tachycardia >90 beats/minute, tachypnoea >20 breaths/minute, leucocytosis >1,200/mm3, <4,000/mm3 or bandemia ≥10%.^18^ In the Sepsis-3, SIRS criteria was considered as a non-specific indicator of dysregulated, life-threatening host response but it may still remain useful for the identification of infection.^18^ Therefore, we used SIRS criteria to analyse the pattern of empirical antibiotic treatment. Because the lactate measurement is limited in primary and secondary hospitals in Vietnam, we defined septic shock in a patient with suspicion of infection and a systolic blood pressure less than 90 mmHg or a mean arterial pressure less than 65 mmHg or requiring administration of vasopressors or treating doctor's clinical judgement.

The quick Sequential related Organ Failure Assessment (qSOFA) score was used to assess the severity of organ dysfunction in all patients. It consists of three components, assigning one point for each: respiratory rate ≥22 breaths per min, systolic blood pressure ≤100 mmHg, and Glasgow Coma Score (GCS) <15.^18^

### Statistical analysis

Data were entered in Epidata (EpiData Association, Odense, Denmark) and analysed using IBM SPSS Statistics for Windows, Version 27.0. Armonk, NY: IBM Corp. Standard descriptive statistics were calculated for categorical (in percentage) and continuous variables (in median and interquartile, IQR). Differences between CCUs in the primary and secondary hospitals were analysed with Pearson chi-square test or Fisher's exact test for categorical variables when appropriated.

Logistic regression was used to identify variables that predict abilities of choosing Watch and Reserve group antibiotics. A previous study in Vietnam have shown that doctors tended to choose a broader spectum for empirical treatment when the patients had more severe illness, older age and medical comorbidities.^19^ An in depth interviews of Vietnamese doctors has shown their considerations on white blood cells, age and underlying disesses when antibiotic prescribing for pneumonia.^20^ Therefore, we chose age, number of comorbidities, SIRS, level of hospital and diagnosis on admission for our multivariable logistic regression model. We used the variance inflation factor (VIF) to test multicollinearity for the model. The VIF values for variables ranged from 1.074 to 1.184. None of the VIF values exceeds 5 and therefore we considered as no collinearity. Differences were considered statistically significant at p values ≤ 0.05.

### Ethics

Eligible patients and/or their relatives were verbally informed about the study. The institutional review board (IRB) in the Hanoi Medical University approved the study (59/GCN-DDNCYSH-DHYHN). The IRB approved a waiver of consent based on the minimal risk to the participants.

## Role of the funding source

The authors did not receive any funds for conducting this study. The corresponding author had full access to all the data in the study and had final responsibility for the decision to submit for publication.

## Results

We enrolled consecutively a total of 1747 adult patients admitted to 51 CCUs, including 36/51 (70.6%) CCUs in primary and 15/51 (29.4%) CCUs secondary hospitals in 5 provinces in Vietnam for a study period of 7 days in each study CCUs from March 2019 to July 2019. The demographics of patients was showed in Table 1. The most common diagnosis on admission were any diagnosis of infection (52.5%, 918/1747). Leading infectious causes of CCU admission were SARI (86.5%, 794/918), abdominal infections (5.2%, 48/918), cardiovascular infections (0.9%, 8/918) and other infection (2.72%, 25/918). Of 794 patients with SARI on admission, exacerbation of COPD was diagnosed in 35.3% (280/794).

**Table 1:** Characteristics of patients admitted to the critical care units.

Characteristics	All patients (n=1747)	CCUs in primary hospitals (n=980)	CCUs in secondary hospitals (n=767)	P value
Age (median, IQR) (years)	68 (55-81)	68 (56-81)	68 (53-80)	0.422
Male gender (%)	988 (56.6%)	531 (54.2%)	457 (59.6%)	0.024
Onset to CCU admissions (median, IQR) (days)	1 (0-3)	1 (0-3)	1 (0-3)	
Number of comorbidity	1177 (67.4%)	681 (69.5%)	496 (64.7%)	0.044
No comorbidity	570 (32.6%)	299 (30.5%)	271 (35.3%)	
One comorbidity	700 (40.1%)	401 (40.9%)	299 (39.0%)	
Two comorbidities	389 (22.3%)	237 (24.2%)	152 (19.8%)	
Three and more comorbidities	88 (5.0%)	43 (4.4%)	45 (5.9%)	
Medical history				
Cardiovascular diseases	689 (39.4%)	404 (41.2%)	285 (37.2%)	0.084
Chronic respiratory disease	584 (33.4%)	396 (40.4%)	188 (24.5%)	<0.001
Diabetes	183 (10.5%)	85 (8.7%)	98 (12.8%)	0.005
Self reported alcoholism	104 (6.0%)	50 (5.1%)	54 (7.0%)	0.089
Chronic liver diseases	80 (4.6%)	39 (4.0%)	41 (5.3%)	0.175
Chronic kidney diseases	71 (4.1%)	29 (3.0%)	42 (5.5%)	0.008
Malignancy	59 (3.4%)	18 (1.8%)	41 (5.3%)	<0.001
Diagnosis at admission				<0.001
SARI	749 (45.4%)	515 (52.6%)	279 (36.4%)	
Non SARI infection	124 (7.1%)	42 (4.3%)	82 (10.7%)	
No infection	829 (47.5%)	423 (43.2%)	406 (52.9%)	
Systemic inflammatory response syndrome (SIRS) (%)	1098 (62.9%)	593 (60.5%)	505 (65.8%)	0.022
Septic shock at admission	124 (7.1%)	38 (3.9%)	86 (11.2%)	<0.001
Quick SOFA score				<0.001
Quick SOFA 0-1	1204 (68.9%)	716 (73.1%)	488 (63.6%)	
Quick SOFA ≥2	543 (31.1%)	264 (26.9%)	279 (36.4%)	
Empirical antibiotic treatment within 24 hours of admission	21 (1.2%)	3 (0.3%)	18 (2.3%)	
No antibiotic therapy	635 (36.3%)	317 (32.3%)	318 (41.5%)	
Single antibiotic therapy	647 (37.0%)	465 (47.4%)	182 (23.7%)	0.001
Dual antibiotic therapy	444 (25.4%)	195 (19.9%)	249 (32.5%)	<0.001
Triple antibiotic therapy	21 (1.2%)	3 (0.3%)	18 (2.3%)	0.068
7-day mortality rate after admission	82 (4.7%)	29 (3.0%)	53 (6.9%)	<0.001

Overall, 63.7% (1112/1747) patients received at least one antibiotic within 24 hours of admission. The overall rate of receiving antibiotics were 63.6% (1112/1747) (663/980, 67.6% and 449/767, 58.5% in patients admitted to CCUs in primary and secondary hospitals, respectively). The rate of antibiotic use in patients with SIRS and any diagnosis of infections were 76.0% (835/1098), 91.9% (828/901) respectively. At the day 7 after CCU admission, 34.0% patient remained hospitalised while 60.2% patients were discharged to home. The overall 7th day mortality rate was 4.7% (82/1747) among patients admitted to CCUs for all causes and was 3.7% (29/794) among patients with SARI. Among patients receiving at least one antibiotic, the proportion of patients receiving any microbiological culture (blood, sputum, urine or other sterile specimen) was 12.9% (144/1112) (31/663 or 4.7% in CCUs in primary hospitals and 121/449 or 26.9% in CCUs in secondary hospitals). The rate of pathogen identification in primary and secondary hospitals were 16.1% (5/31) and 42/121 (34.7%), respectively. No susceptibility results were further collected.

There were 31.5% (267/846) patients without diagnosis of infection in the medical notes received any antibiotic, while 6.8% (62/901) patients with diagnosis of any bacterial infection received no antibiotic within 48 hours of admission. There was a total of 72 different antibiotics used over all CCUs and (Table 3) represented for essential antibiotics by WHO Model List of Essential Medicines. Among patients received at least one antibiotic, the most common prescribed antibiotics classes were the 3^rd^ generation cephalosporins, fluoroquinolones and penicillins with beta lactamase inhibitors (Table 2). Table 3 stratified available antibiotic by chemical substance.

**Table 2:** Empirical antibiotic prescriptions in patients receiving at least one antibiotics on admission to CCUs.

Antibiotics prescription	All patients (n=1112)	CCUs in primary hospitals (n=663)	CCUs in secondary hospitals (n=449)	p values
J01DD_Third generation cephalosporins	671 (60.3%)	424 (64.0%)	247 (55.0%)	0.003
J01MA_Fluoroquinolones	350 (31.5%)	145 (21.9%)	205 (45.7%)	<0.001
J01CR_Combinations of penicillins, incl. beta lactamase inhibitors	165 (14.8%)	90 (13.6%)	75 (16.7%)	0.150
J01DC_Second generation cephalosporins	120 (10.8%)	101 (15.2%)	19 (4.2%)	<0.001
J01GB_Other aminoglycosides	104 (9.4%)	66 (10.0%)	38 (8.5%)	0.402
J01DH_Carbapenems	67 (6.0%)	1 (0.2%)	66 (14.7%)	<0.001
J01XD_Imidazole derivatives	44 (4.0%)	15 (2.3%)	29 (6.5%)	<0.001
J01XA_Glycopeptide antibiotics	14 (1.3%)	0 (0.0%)	14 (3.1%)	<0.001
J01CA_Penicillins with extended spectrum	13 (1.2%)	7 (1.1%)	6 (1.3%)	0.669
J01DB_First generation cephalosporins	12 (1.1%)	6 (0.9%)	6 (1.3%)	0.495
J01DE_Fourth generation cephalosporins	10 (0.9%)	0 (0.0%)	10 (2.2%)	<0.001
J01FA_Macrolides	8 (0.7%)	6 (0.9%)	2 (0.4%)	0.374
J01EA_Combinations of sulfonamides and trimethoprim, including derivatives	5 (0.4%)	1 (0.2%)	4 (0.9%)	0.070
J01FF_Lincosamides	4 (0.4%)	0 (0.0%)	4 (0.9%)	0.015
J01XX_Other antibiotics	3 (0.3%)	0 (0.0%)	3 (0.7%)	0.035
J01XB_Polymyxins	3 (0.3%)	0 (0.0%)	3 (0.7%)	0.035
J01MB_Other quinolones	2 (0.2%)	1 (0.2%)	1 (0.2%)	0.781
J01CE_Beta lactamase sensitive penicillins	1 (0.1%)	1 (0.2%)	0 (0.0%)	0.410

**Table 3:** Frequency of antibiotic use as empirical therapy within 24 hours of CCUs admission.

Antibiotics by ACT	All patients (n=1112)	CCUs in primary hospitals (n=663)	CCUs in secondary hospitals (n=449)	p values
J01DD01_cefotaxime	248 (22.3%)	178 (26.8%)	70 (15.6%)	<0.001
J01MA12_levofloxacin	211 (19.0%)	73 (11.0%)	138 (30.7%)	<0.001
J01DD02_ceftazidime	120 (10.8%)	104 (15.7%)	16 (3.6%)	<0.001
J01DD04_ceftriaxone	113 (10.2%)	62 (9.4%)	51 (11.4%)	0.785
J01DD12_cefoperazone	104 (9.4%)	24 (3.6%)	80 (17.8%)	<0.001
J01DC02_cefuroxime	100 (9.0%)	96 (14.5%)	4 (0.9%)	<0.001
J01MA02_ciprofloxacin	96 (8.6%)	49 (7.4%)	47 (10.5%)	0.305
J01CR01_ampicillin and beta-lactamase inhibitor	89 (8.0%)	55 (8.3%)	34 (7.6%)	0.266
J01DD07_ceftizoxime	52 (4.7%)	51 (7.7%)	1 (0.2%)	<0.001
J01GB03_gentamicin	50 (4.5%)	50 (7.5%)	0 (0.0%)	<0.001
J01CR02_amoxicillin and beta-lactamase inhibitor	42 (3.8%)	35 (5.3%)	7 (1.6%)	<0.001
J01DH51_imipenem and cilastatin	41 (3.7%)	0 (0.0%)	41 (9.1%)	<0.001
J01GB06_amikacin	41 (3.7%)	4 (0.6%)	37 (8.2%)	<0.001
J01MA14_moxifloxacin	40 (3.6%)	22 (3.3%)	18 (4.0%)	0.888
J01CR05_piperacillin and beta-lactamase inhibitor	34 (3.1%)	0 (0.0%)	34 (7.6%)	<0.001
J01XD01_metronidazole	34 (3.1%)	15 (2.3%)	19 (4.2%)	0.155
J01DD62_cefoperazone and beta-lactamase inhibitor	28 (2.5%)	1 (0.2%)	27 (6.0%)	<0.001
J01DH02_meropenem	26 (2.3%)	1 (0.2%)	25 (5.6%)	<0.001
J01DC01_cefoxitin	14 (1.3%)	0 (0.0%)	14 (3.1%)	<0.001
J01GB01_tobramycin	12 (1.1%)	11 (1.7%)	1 (0.2%)	0.013
J01XD02_tinidazole	10 (0.9%)	0 (0.0%)	10 (2.2%)	<0.001
J01DE01_cefepime	9 (0.8%)	0 (0.0%)	9 (2.0%)	0.001
J01XA01_vancomycin	8 (0.7%)	0 (0.0%)	8 (1.8%)	0.001
J01XA02_teicoplanin	6 (0.5%)	0 (0.0%)	6 (1.3%)	0.006
J01DD08_cefixime	6 (0.5%)	3 (0.5%)	3 (0.7%)	1.000
J01EE01_sulfamethoxazole and trimethoprim	5 (0.4%)	1 (0.2%)	4 (0.9%)	0.175
J01CA04_amoxicillin	5 (0.4%)	4 (0.6%)	1 (0.2%)	0.393
J01FA10_azithromycin	5 (0.4%)	5 (0.8%)	0 (0.0%)	0.071
J01CA12_piperacillin	4 (0.4%)	0 (0.0%)	4 (0.9%)	0.037
J01DB05_cefadroxil	4 (0.4%)	0 (0.0%)	4 (0.9%)	0.037
J01FF01_clindamycin	4 (0.4%)	0 (0.0%)	4 (0.9%)	0.037
J01CA01_ampicillin	4 (0.4%)	3 (0.5%)	1 (0.2%)	0.636
J01DC03_cefamandole	4 (0.4%)	4 (0.6%)	0 (0.0%)	0.136
J01XB01_colistin	3 (0.3%)	0 (0.0%)	3 (0.7%)	0.084
J01FA09_clarithromycin	3 (0.3%)	1 (0.2%)	2 (0.4%)	0.585
J01DB12_ceftezole	3 (0.3%)	2 (0.3%)	1 (0.2%)	1.000
J01XX08_linezolid	2 (0.2%)	0 (0.0%)	2 (0.4%)	0.193
J01DB01_cefalexin	2 (0.2%)	1 (0.2%)	1 (0.2%)	1.000
J01MA01_ofloxacin	2 (0.2%)	1 (0.2%)	1 (0.2%)	1.000
J01MB02_nalidixic acid	2 (0.2%)	1 (0.2%)	1 (0.2%)	1.000
J01DB04_cefazolin	2 (0.2%)	2 (0.3%)	0 (0.0%)	0.507
J01DC09_cefmetazole	1 (0.1%)	0 (0.0%)	1 (0.2%)	0.439
J01DE02_cefpirome	1 (0.1%)	0 (0.0%)	1 (0.2%)	0.439
J01MA09_sparfloxacin	1 (0.1%)	0 (0.0%)	1 (0.2%)	0.439
J01XX01_fosfomycin	1 (0.1%)	0 (0.0%)	1 (0.2%)	0.439
J01CE01_benzylpenicillin	1 (0.1%)	1 (0.2%)	0 (0.0%)	1.000
J01DB03_cefalotin	1 (0.1%)	1 (0.2%)	0 (0.0%)	1.000
J01DC04_cefaclor	1 (0.1%)	1 (0.2%)	0 (0.0%)	1.000
J01DD13_cefpodoxime	1 (0.1%)	1 (0.2%)	0 (0.0%)	1.000
J01GB07_netilmicin	1 (0.1%)	1 (0.2%)	0 (0.0%)	1.000

**Figure 1:**
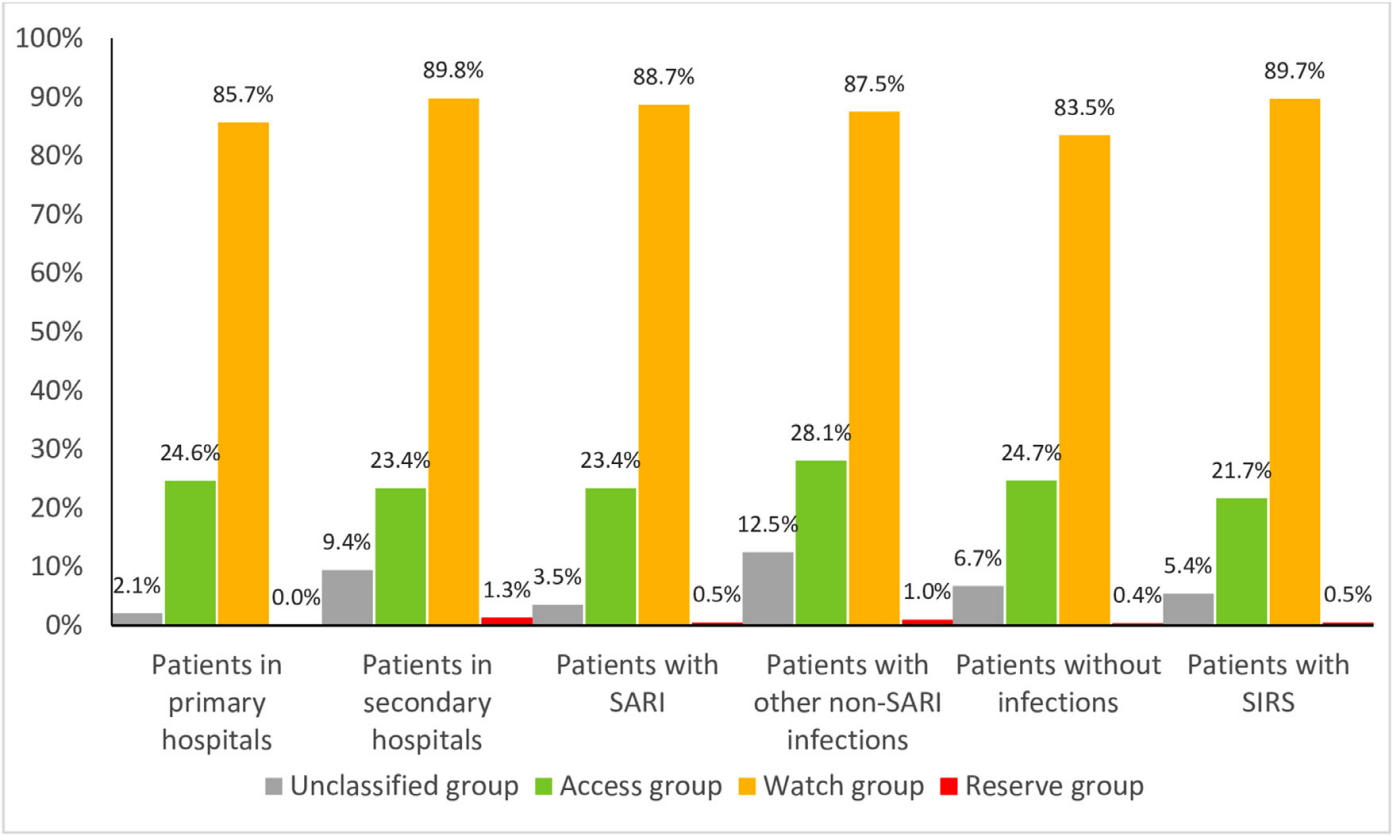
Empirical antibiotic regimens within 24 hours of admission to CCUs by AWaRe classification.

The proportion of any Access group, Watch group, Reserve group and non-recommended antibiotics were 24.1% (268/1112), 87.3% (971/1112), 0.54% (6/1112) and 5.0% (56/1112), respectively (Table 2 and Fig 1). All 6 patients who received Reserve group antibiotics in secondary hospital CCUs had the diagnosis of SARI (4 patients) and septic shock (1 patients).

Among the 1112 patients who received any antibiotics, 647 (37.0%) were treated with mono therapy, 444 (25.4%) received dual therapy and 21 (1.2%) received triple therapy (Table 1). The rates of antibiotic combination treatment (dual and triple therapy) were lower in CCUs in primary hospitals vs secondary hospitals (198/980, 20.2% vs 267/767, 34.8%, p<0.001), higher in patients with SARI vs other infections (319/866, 40.1% vs 53/124, 42.7%, p<0.001), and higher in patients with qSOFA ≥2 vs qSOFA< 2 (205/543, 37.8% vs 260/1204, 21.6%, p<0.001). The antibiotic regimens were showed in the Table 4.

**Table 4:** Empirical antibiotic regimens within 24 hours of admission to CCUs by the Anatomical Therapeutic Chemical (ATC) Index.

Antibiotic regimens	All patients (n=1112)	Patients with SARI (n=749)	Patients with other infection (n=96)	Patients without infection (n=267)
Mono antibiotic therapy	647 (58.2%)	430 (57.4%)	43 (44.8%)	174 (65.2%)
J01DD_Third generation cephalosporins	408 (63.1%)	290 (67.4%)	22 (51.2%)	96 (55.2%)
J01CR_Combinations of penicillins, incl. beta lactamase inhibitors	85 (13.1%)	60 (14.0%)	4 (9.3%)	21 (12.1%)
J01DC_Second generation cephalosporins	77 (11.9%)	55 (12.8%)	2 (4.7%)	20 (11.5%)
J01MA_Fluoroquinolones	42 (6.5%)	17 (4.0%)	9 (20.9%)	16 (9.2%)
J01DB_First generation cephalosporins	8 (1.2%)	2 (0.5%)	3 (7.0%)	3 (1.7%)
J01XD_Imidazole derivatives	8 (1.2%)	0 (0.0%)	1 (2.3%)	7 (4.0%)
J01CA_Penicillins with extended spectrum	6 (0.9%)	0 (0.0%)	1 (2.3%)	5 (2.9%)
Other mono therapy	13 (2.0%)	6 (1.4%)	1 (2.3%)	6 (3.4%)
**Dual antibiotic therapy**	**444 (39.9%)**	**310 (41.4%)**	**48 (50.0%)**	**86 (32.2%)**
J01DD_Third generation cephalosporins and J01MA_Fluoroquinolones	163 (36.7%)	115 (37.1%)	15 (31.3%)	33 (38.4%)
J01DD_Third generation cephalosporins and J01GB_Other aminoglycosides	57 (12.8%)	48 (15.5%)	5 (10.4%)	4 (4.7%)
J01CR_Combinations of penicillins, incl. beta lactamase inhibitors and J01MA_Fluoroquinolones	54 (12.2%)	41 (13.2%)	4 (8.3%)	9 (10.5%)
J01DH_Carbapenems and J01MA_Fluoroquinolones	36 (8.1%)	19 (6.1%)	7 (14.6%)	10 (11.6%)
J01DC_Second generation cephalosporins and J01MA_Fluoroquinolones	28 (6.3%)	24 (7.7%)	1 (2.1%)	3 (3.5%)
J01DD_Third generation cephalosporins and J01XD_Imidazole derivatives	23 (5.2%)	4 (1.3%)	10 (20.8%)	9 (10.5%)
J01CR_Combinations of penicillins, incl. beta lactamase inhibitors and J01GB_Other aminoglycosides	16 (3.6%)	16 (5.2%)	0 (0.0%)	0 (0.0%)
J01DH_Carbapenems and J01GB_Other aminoglycosides	8 (1.8%)	7 (2.3%)	0 (0.0%)	1 (1.2%)
J01DE_Fourth generation cephalosporins and J01MA_Fluoroquinolones	5 (1.1%)	3 (1.0%)	1 (2.1%)	1 (1.2%)
Other dual therapy	54 (12.2%)	33 (10.6%)	5 (10.4%)	16 (18.6%)
**Triple and quadruple antibiotic therapy**	**21 (1.9%)**	**9 (1.2%)**	**5 (5.2%)**	**7 (2.6%)**
J01DD_Third generation cephalosporins, J01MA_Fluoroquinolones and other	6 (28.6%)	1 (11.1%)	1 (20.0%)	4 (57.1%)
J01DH_Carbapenems, J01MA_Fluoroquinolones and other	5 (23.8%)	2 (22.2%)	1 (20.0%)	2 (28.6%)
Carbapenems and 2 others	5 (23.8%)	2 (22.2%)	3 (60.0%)	0 (0.0%)
J01CR_Combinations of penicillins, incl. beta lactamase inhibitors, J01MA_Fluoroquinolones and other	3 (14.3%)	2 (22.2%)	0 (0.0%)	1 (14.3%)
Others	2 (9.5%)	2 (22.2%)	0 (0.0%)	0 (0.0%)

Independent factors associated with using Watch and/or Reserve groups were patient's age (aOR 1.01 per 1-year increment, 95%CI 1.00-1.02) and SIRS (aOR 2.1, 95%CI 1.38-3.2) (Table 5).

**Table 5:** Logistic regression analysis of factors associated with empirical therapy of Watch and Reverse group antibiotics on admission.

	Adjusted OR (95% CI)	P value
Age (years) (1-yr. increment)	1.01 (1.00-1.02)	0.014
Number of comorbidities		
No comorbidity	1	
1 comorbidity	0.66 (0.42-1.04)	0.076
At least 2 comorbidities	1.28 (0.74-2.22)	0.384
quick Sequential Organ Failure Assessment (qSOFA)		
qS OFA 0-1	1	
qSOFA 2-3	0.80 (0.53-1.22)	0.299
Systemic Inflammatory Response Syndrome (SIRS)		
Without SIRS on admission	1	
With SIRS on admission	2.10 (1.38-3.20)	0.001
Diagnosis on admission		
No diagnosis of any type of infection	1	
Non-respiratory infection	1.39 (0.69-2.81)	0.363
SARI	1.50 (0.99-2.29)	0.058
Level of hospitals		
Primary hospitals	1	
Secondary hospitals	1.50 (1.00-2.23)	0.05

## Discussion

To the best of our knowledge, our study represents the first effort to describe the initially empirical antibiotic therapy in CCUs in Vietnam, a country with a high burden of antibiotic drug resistant.

Our study was completed 5 months before the COVID-19 that was reported in Vietnam in January 2020^20^ and was declared as a global pandemic by WHO in March 2020.^21^ The high proportion of SARI cases in CCUs in our study had shown an existing burden on the healthcare system in Vietnam and the current issues of SARI case management had indicated a possibility of overwhelming demands of intensive care services if more SARI related cases would have occurred. At the global level, lower respiratory infections ranked the second as a causes of disease burden (in disability-adjusted life year, DALY) and ranked the fourth as a cause of deaths.^22^ The percentage of SARI among CCUs admission in our study were much higher than other studies in high income countries. In high income settings, sepsis was presented in 11-28% on admissions to ICUs in which respiratory infections was still the most common cause of sepsis (28-68%).^23^, ^24^, ^25^ In a study of sepsis in Southeast Asia (including Vietnam), acute respiratory infection was the most frequent diagnosis in adult patients with sepsis (53%).^26^ At the time of this study, there were no reported outbreaks of respiratory infections in the country. However, of note, the circulation of influenza was known as year-round in the country; potentially with peaks in June to August and in December to January in northern Vietnam.^27^

In Vietnamese guideline on antibiotic use in 2015, antibiotics recommended for moderate pneumonia were amoxicillin plus clarithromycin or benzylpenicillin plus clarithromycin or β-Lactam (cefotaxime, ceftriaxone) or ampicillin-sulbactam plus macrolide or fluroquinolone. For severe pneumonia, it was recommended to start^1^ amoxicillin/clavulanic acid plus clarithromycin or^2^ benzylpenicillin plus quinolone or^3^ a third cephalosporin plus clarithromycin.^28^ We found a quite variations of antibiotic prescriptions by in CCUs. The reasons may be attributed to the lack of timely updates, not supported by local susceptibility data, and not reflecting doctors’ behaviours or comments in the national guideline. Consequently, in a survey in 1280 health professionals in Vietnam, empirical antibiotic selection was decided by infection source and diseases severity.^19^ The large numbers and complexity of available antibiotics may create challenges for clinicians and pharmacists in choice of empirical therapy. Additionally, the lack of confirmatory laboratory capacity, such as bacterial cultures and PCR for viral etiologies, is still an obstacle to the implementation of antimicrobial stewardship programme in in resource constraint countries.

In a previous study, the percentages of antibiotic purchased in Access, Watch and Reverse groups in health care facilities in Viet Nam, were 47.2%, 52.4% and 0.1% respectively.^29^ The most commonly used antibiotics in CCUs in provincial and district general hospitals were cephalosporins, penicillins, aminoglycosides and imidazole^30^ while they were the third generation cephalosporins, fluoroquinolones, and carbapenems in the CCUs setting.^31^ The high frequency of Watch and Reserve group antibiotics (87.3% and 0.54% respectively) in our study indicated the strategy of early administration of broad-spectrum antibiotic in CCUs, and therefore, the surveillance on antibiotic consumption and patterns of prescription is particularly important to ensure a rational antibiotic use.

Our study had some limitations. Firstly, we did not collect data to distinguish between antibiotic treatments and perioperative antibiotic prophylaxis. However, we considered the proportion of prescription for preoperative antibiotic prophylaxis was small because of the low rate of surgery within 24 hours of admission in our study participants (27/1747 or 1.5%). Secondly, because of the short-period observational study design, there may be some bias in evaluation of causes of admission. Additionally, the majority of initial diagnosis of infection were clinically made, partly related to the lack of rapid diagnostics whilst the empirical antibiotic prescribing decisions were influenced by doctors’ experiences and by level of hospitals. It makes the interpretation of empirical antibiotic choice difficult and must be related to the current burden of antibiotic resistant pathogens in community and in a particular CCU. Thirdly, due to the limitations of study design, data collection and low frequency of microbiological culture, we were unable to evaluate the necessity and appropriateness of antibiotic treatment in our study patients. Further studies are required to evaluate the compliance with antimicrobial treatment guidelines for empirical antibiotic selection and rational antibiotic use in relation to diagnosis and microbiological findings in Vietnam.

In conclusion, there was an over prescription of broad spectrum antibiotic and high frequency of antibiotic combination for all causes of admission to CCUs in primary and secondary hospitals in Vietnam. It is crucial to implement the surveillance of antibiotic use in CCUs and establish a protocol the empirical antibiotic treatment to improve overall SARI patients’ outcome.

### Contributors

VQD, SO supervised the project implementation, including designing the study and analysing the data. VQD, TTD reviewed and classified diagnosis on admission. SO, VQH, KBG and VQD collected data and monitor the study. VQD, TTD has consolidated and draughted the first report. All authors involved to the acquisition and interpretation of data. All authors contributed to and approved the final report.

## Declaration of Competing Interest

We declare no competing interest.

## Data Availability

All data requests will be considered by the corresponding author for approval.

## Appendix Supplementary materials

Image, application 1

Image, application 2
