# Supplementary material for: Antibiotic use for empirical therapy in the critical care units in primary and secondary hospitals in Vietnam: a multicenter cross-sectional study
Source: Lancet Reg Health West Pac. 2021 Nov 3;18:100306. doi: 10.1016/j.lanwpc.2021.100306 (PMC8669321; doi:10.1016/j.lanwpc.2021.100306)
Supplement: Supplementary file 1 [file mmc1.docx]

# This translation in ****Vietnamese**** was submitted by the authors and we reproduce it as supplied. It has not been peer reviewed. Our editorial processes have only been applied to the original abstract in English, which should serve as reference for this manuscript.

# Tóm tắt nghiên cứu

## Tổng quan

Tỉ lệ nhiễm trùng cao ở những bệnh nhân nhập khoa cấp cứu có liên quan tới tình trạng sử dụng kháng sinh phổ biến, đặc biệt là các kháng sinh phổ rộng. Nghiên cứu này mô tả việc sử dụng kháng sinh ở các khoa cấp cứu ở các bệnh viện tuyến huyện và tuyến tỉnh tại Việt Nam, nơi có tỉ lệ kháng kháng sinh cao.

## Phương pháp

Đây là nghiên cứu quan sát trong 7 ngày ở 71 khoa cấp cứu tại 5 tỉnh tại Việt Nam từ tháng 3 đến tháng 7 năm 2019. Những bệnh nhân từ 18 tuổi trở lên nhập khoa cấp cứu được thu tuyển liên tục. Chúng tôi thu thập thông tin về nhân khẩu học, chẩn đoán ban đầu và điều trị kháng sinh trong vòng 24 giờ đầu nhập viện. Kháng sinh được phân loại theo hệ thống Anatomical Therapeutic Chemical (ATC) Index và phân nhóm Tiếp cận (Access), Theo dõi (Watch), Dự trữ (Reserve) (AWaRe) theo tổ chức y tế thế giới (WHO) năm 2019.

## Kết quả

Trong số 1747 bệnh nhân được thu tuyển, kháng sinh theo kinh nghiệm được sử dụng ở 1112 (63.6%) bệnh nhân. Kháng sinh thường được kê nhất là cefotaxime (22.3%), levofloxacin (19%) và ceftazidime (10.8%). Các kháng sinh được kê cho 31.5% bệnh nhân không có chẩn đoán nhiễm trùng. Kháng sinh nhóm Theo dõi và/hoặc Dự trữ được kê cho 87.3% số bệnh nhân và liên quan tới tuổi (aOR 1.01 cho mỗi tuổi tăng thêm, 95%CI 1.00-1.02) và có SIRS khi nhập viện (aOR 2.1, 95%CI 1.38-3.2).

## Phiên giải

Chúng tôi quan sát thấy tỉ lệ sử dụng kháng sinh cao và có nhiều khác biệt về sử dụng kháng sinh theo kinh nghiệm ở các khoa cấp cứu tại Việt Nam. Điều đó nhấn mạnh tầm quan trọng của việc giám sát sử dụng kháng sinh ở các khoa cấp cứu.

# Kinh phí

Các tác giả không nhận bất kỳ kinh phí nào để triển khai nghiên cứu này
